# The Influences of Nb Microalloying and Grain Refinement Thermal Cycling on Microstructure and Tribological Properties of Armor Steel

**DOI:** 10.3390/ma16237485

**Published:** 2023-12-02

**Authors:** Mervat Youssef, Eman H. El-Shenawy, Wael Khair-Eldeen, Tadaharu Adachi, Adel Nofal, Mohsen A. Hassan

**Affiliations:** 1 School of Innovative Design Engineering (IDE), Egypt-Japan University of Science and Technology (E-JUST), Alexandria 21934, Egypt; wael.khaireldin@ejust.edu.eg; 2 Metal Casting Department, Manufacturing Technology Institute, Central Metallurgical Research and Development Institute (CMRDI), Cairo 11912, Egypt; adelnofal@hotmail.com; 3 Plastic Deformation Department, Metal Technology Institute, Central Metallurgical Research and Development Institute (CMRDI), Cairo 11912, Egypt; dremanelshenawy@cmrdi.sci.eg; 4 Department of Mechanical Engineering, Toyohashi University of Technology (TUT), Aichi 441-8122, Japan; adachi.tadaharu.or@tut.jp

**Keywords:** Armor Steel, Nb microalloying, heat treatment, grain refinement, surface texture, Abbott–Firestone curve, tribology, microhardness

## Abstract

This study aims to investigate the combined effect of niobium (Nb) microalloying and austenite grain refinement, using a specific heat treatment cycle, on the microstructure and tribological properties of two newly developed armor steels prepared on a laboratory scale. In this work, Nb addition and thermal cycling were utilized for grain refinement and enhancement of the mechanical properties of investigated alloys, to provide improved protection via lightweight armor steel components with a high strength-to-weight ratio. The kinetics of transformation of the developed Armor alloys were studied using JMATPro version 13.2. The samples were subjected to two austenitizing temperatures, 1000 °C and 1100 °C, followed by 4 min of holding time and three consecutive thermal and rapid-quenching processes from 900 °C to room temperature. Scanning electron microscopy with energy dispersive X-ray analysis (SEM-EDX) was employed to analyze the microstructure, which primarily consists of four types of martensite: short and long lath martensite, blocky martensite, and equiaxed martensite. Additionally, a small percentage (not exceeding 3%) of carbide precipitates was observed. The wear characteristics of the investigated alloys were evaluated using a pin-on-disc tribometer. The results demonstrate that alloying with Nb and grain refinement using a thermal cycle significantly reduce the wear rate.

## 1. Introduction

Worldwide infrastructure and the special-purpose vehicles utilized by civilian and military forces extensively rely on advanced protective materials, including armor steels, composites, and ceramics, to ensure protection against various threats [1,2,3,4]. Moreover, the growing demands for improved fuel efficiency, reduced greenhouse gas emissions, and enhanced safety and crash-resistant performance have made high-strength lightweight vehicles a top priority for manufacturers [5,6].

Commercial quenched-and-tempered armor steel (≈500 HBW class) is widely recognized as a conventional armor steel that offers a combination of high strength, hardness, toughness, exceptional ballistic performance, superior machinability, ease of heat treatment, and weldability [7,8,9]. It is a low-alloy hot-rolled steel that is available in thicknesses ranging from 3.0 to 80.0 mm, with a hardness of 500 HBW and a tensile strength of 1750 MPa. The final plates and sheets undergo a heat treatment procedure involving austenitizing, quenching, and tempering stages [10].

The most effective methods for enhancing and controlling the final properties of armor steels involve alloying additions, heat treatment, and thermomechanical processing [11,12]. It is widely reported that the grain size of austenite plays a crucial role in improving the hardenability, tensile strength, and toughness properties of steel [13,14,15,16,17,18]. Therefore, obtaining a small prior austenite grain size is essential in manufacturing Armor steel to achieve the desired structures of short lathes and equiaxed martensite after quenching.

However, it is important to carefully control microalloying additions to avoid detrimental effects such as segregation, retained austenite, and the formation of secondary phases, including inclusions, primary carbides, and nitrides [19,20].

Boron (B) is a traditional microalloying element that is widely known for significantly improving steel’s hardenability, toughness, and tensile strength by segregating to the grain boundaries of distorted austenite regions [21,22]. Due to the atomic size ratio of boron to iron, which is 0.66 for interstitial alloying and 0.85 for substitutional alloying in steel, boron exhibits low solubility in austenite, effectively hindering austenite grain growth [19,20]. However, studies have reported negative effects of boron, including the potential promotion of austenite grain growth and a decrease in grain growth starting temperature [23,24]. This phenomenon is attributed to the formation of boron nitride particles, which have a lesser inhibiting effect on grain growth. The addition of nitride-forming elements such as Nb, Cr, V, Mo, W, Al, and Ti in appropriate amounts can prevent the formation of boron nitrides and enhance the efficiency of boron as a hardenability intensifier [25,26].

Furthermore, Nb microalloying plays a significant role in refining the austenite structure of steel. High-strength steels commonly contain low amounts of microalloying elements, including Ti, V, and Nb, as they exhibit a strong affinity for carbon and nitrogen. Studies have reported that Nb and V carbonitrides of types M(C, N) and M2(C, N) effectively control austenite growth and improve both toughness and tensile strength [27,28]. Moreover, for various steel grades, Nb is selected to refine the austenite structure during hot deformation processes [29,30]. Due to Nb’s larger atomic size compared to iron (Fe), it exerts a strong solute drag effect on the grain boundaries of austenite γ, resulting in a significant retardation of recrystallization. Additionally, unprecipitated Nb delays phase transitions to lower temperatures, leading to increased hardening and toughening during the transition [31,32].

The Abbott–Firestone technique was used to quantitively measure the surface textural quality and phase distribution of the investigated alloys. This surface characterization technique highlights the dependence of tribological properties on surface topography [33].

As mentioned earlier, the crucial requirements for components that will be used in military and high abrasion-resistant applications are sufficient strength and resistance to wear. However, limited research is available in the literature regarding the tribological characteristics of Armor steels. Similar studies in high-strength steels [34,35,36] have focused on wear-related issues. The fluctuation of lost volume, or wear resistance, is influenced by various factors, including applied load, component geometry, the relative motion of surfaces, sliding distance, surface roughness, chemical composition, the microstructure of the alloys, operating temperature, lubrication, and the impact of vibration on the frictional behavior of the alloys. For instance, Saxena et al. [37] conducted dry sliding wear tests on shielded metal arc welded (SMAW) joints made of commercial quenched-and-tempered armor steel (~500 HBW class). The tests involved varying an applied force up to 120 N and sliding distances up to 6 km. During the experiments, a pin-on-disk tribometer was utilized to maintain a consistent sliding velocity. The findings indicate that the base metal showed enhanced wear resistance compared to the weld joints. This observation could be attributed to microstructure evolution, whereby the presence of martensite lath in an acicular morphology, accompanied by coarse and fine precipitates, contributes to improved tribological behavior.

This study investigates the influence of Nb microalloying and heat treatment on the microstructure, microhardness, and tribological behavior of two newly developed armor steels prepared on a laboratory scale. The impact of Nb addition on the morphology was examined through a scanning electron microscope (SEM) with EDX. Furthermore, the alloy design was optimized using JMATPro to determine the combined effect of Nb microalloying and heat treatment on hardness, transformation temperatures, and phase volume fraction in the developed Armor alloys. The actual transformation temperatures were determined through differential scanning calorimetry analysis (DSC). Additionally, the surface texture of Nb microalloyed Armor Steel was assessed using an Abbott–Firestone curve, generated via a MATLAB code. Finally, the wear characteristics of the investigated alloys were evaluated using a pin-on-disc tribometer.

## 2. Materials and Methods

### 2.1. Melting, Casting, and the Hot-Forging Process

This study involved two Armor steel alloys with different Nb and B contents. The melting process was conducted using a 300 kg medium-frequency quartzite-lined induction furnace. To achieve the desired chemical composition shown in Table 1, high-purity steel scrap, carburizer, ferroalloys, and pure metals were employed. It is important to note that the alloying of molten steel with FeB and FeNb necessitates specific control parameters [38,39,40,41]. To prevent segregation, coarse inclusions, and undesired precipitation in the liquid steel and to enhance its recovery in solidified castings, FeNb was added to the steel melt in the form of small particles during the final step of the melting process. Following the complete melting of the alloying additions, the molten steel was poured into green sand molds in the shape of keel blocks measuring 200 × 150 × 25 mm. The chemical composition of all samples was determined using a spectrometry device, the Oxford FOUNDRY-MASTER Pro (Hitachi Ltd., Tokyo, Japan).

The as-cast plates were annealed at 950 °C in a heat-resistance furnace and held at this temperature for 60 min. Subsequently, the annealed plates were hot-forged into round bars with a diameter of 25 mm, using a 250 kg hammer machine. Finally, the forged bars were water-quenched to room temperature.

### 2.2. Simulation Software

JMATPro version 13.2, a simulation software, was utilized to calculate the transformation temperatures and theoretically predict mechanical properties.

### 2.3. Heat Treatment Process

Various heat treatment procedures were employed on the Nb microalloyed Armor steels to achieve different martensite morphologies. The heat treatment cycle was initiated by heating the samples above the Ac3 temperature. Two austenitizing temperatures, 1000 °C and 1100 °C, were chosen, and the samples were held at these temperatures for 4 min. This was followed by a second batch of heat treatment, involving three successive rapid heating and quenching treatments from 900 °C to room temperature. These treatments aimed to enhance the austenite nucleation and ultimately refine the final microstructure. All heat treatment cycles were performed using a quenching dilatometer, specifically the LINSEIS L78/RITA model (Linseis Messgeraete GmbH, Selb, Germany). Cylindrical specimens measuring 3 mm in diameter and 10 mm in length were machined from the forged bars. The heat treatment cycles were conducted under a vacuum of 5 × 10^−2^ Pa using a high-frequency induction system, and the cooling stages were carried out using high-purity helium gas.

### 2.4. Differential Scanning Calorimetry (DSC) Analysis

Differential scanning calorimetry (DSC) analysis was performed to measure and identify the phase transitions in the investigated alloys. A LINSEIS STA PT1600/1000/LT device (Linseis Messgeraete GmbH, Selb, Germany), equipped with an Al_2_O_3_ reference crucible (mass/mg: 18,000), was used for this analysis. Samples were heated to 1200 °C at a heating rate of 10 °C/min and then cooled to room temperature (RT) at a cooling rate of 10 °C/min in an argon gas environment.

### 2.5. Thin-Film X-ray Diffraction (XRD)

A D8 DISCOVER Plus X-ray diffractometer (XRD) (Bruker, Billerica, MA, USA) was employed to detect the phases and determine their crystal structures. Sample preparation for XRD analysis involved grinding and polishing the surface. The prepared samples were subjected to XRD analysis using a diffractometer with CuKα radiation (λ = 0.15406, 40 KV, 30 mA). Measurements were taken within an angle (2θ) range of 10° to 100° at a scanning speed of 0.2 s/step.

### 2.6. Metallography Characterizations

Metallographic characterization was conducted using scanning electron microscopy (SEM) via a JEOL JSM-6010LV instrument (JEOL, Tokyo, Japan). The samples were etched with 3% Nital solution. Additionally, a 3D laser scanning microscope (Keyence VK-X100) (Keyence Corporation of America, Elmwood Park, NJ, USA) analyzed the worn surfaces after tribological testing.

### 2.7. Microhardness Measurements

Microhardness testing was performed using a Shimadzu HMV-2 microhardness tester (SHIMADZU, Kyoto, Japan), based on the Vickers method. A load of 4.903 N (HV 0.5) was applied for 10 s, following the ASTM E384-22 [42] guidelines.

### 2.8. Tribological Investigations

Tribological investigations were carried out using a pin-on-disk tribometer type (T-01 M). The counter disk was made of hard steel (65 HRC), while the pin was a cylindrical specimen fabricated from the developed Armor alloys, with dimensions of 10 mm in length and 3 mm in diameter. The wear test machine was set to a loading condition of 80 N, a rotating speed of 400 rpm, and a sliding distance of 1200 m. Under these operating conditions, the sliding velocity was 0.67 m/s during the 30 min of testing, and the pin track radius was 16 mm. Prior to the test, all specimens were carefully ground and cleaned to ensure conformal contact between the pin and the disk.

Furthermore, debris was removed from the test sample to ensure accurate measurements after completing the test. The tests were conducted at room temperature, and the temperature rise and friction coefficient were monitored and recorded during the trial. These tribological investigations adhered to the ASTM G 99-05 [43] and DIN 50324-07 standards [44].

## 3. Results and Discussion

### 3.1. Effect of Cooling Rate on Phases and Hardness

**Figure 1:**
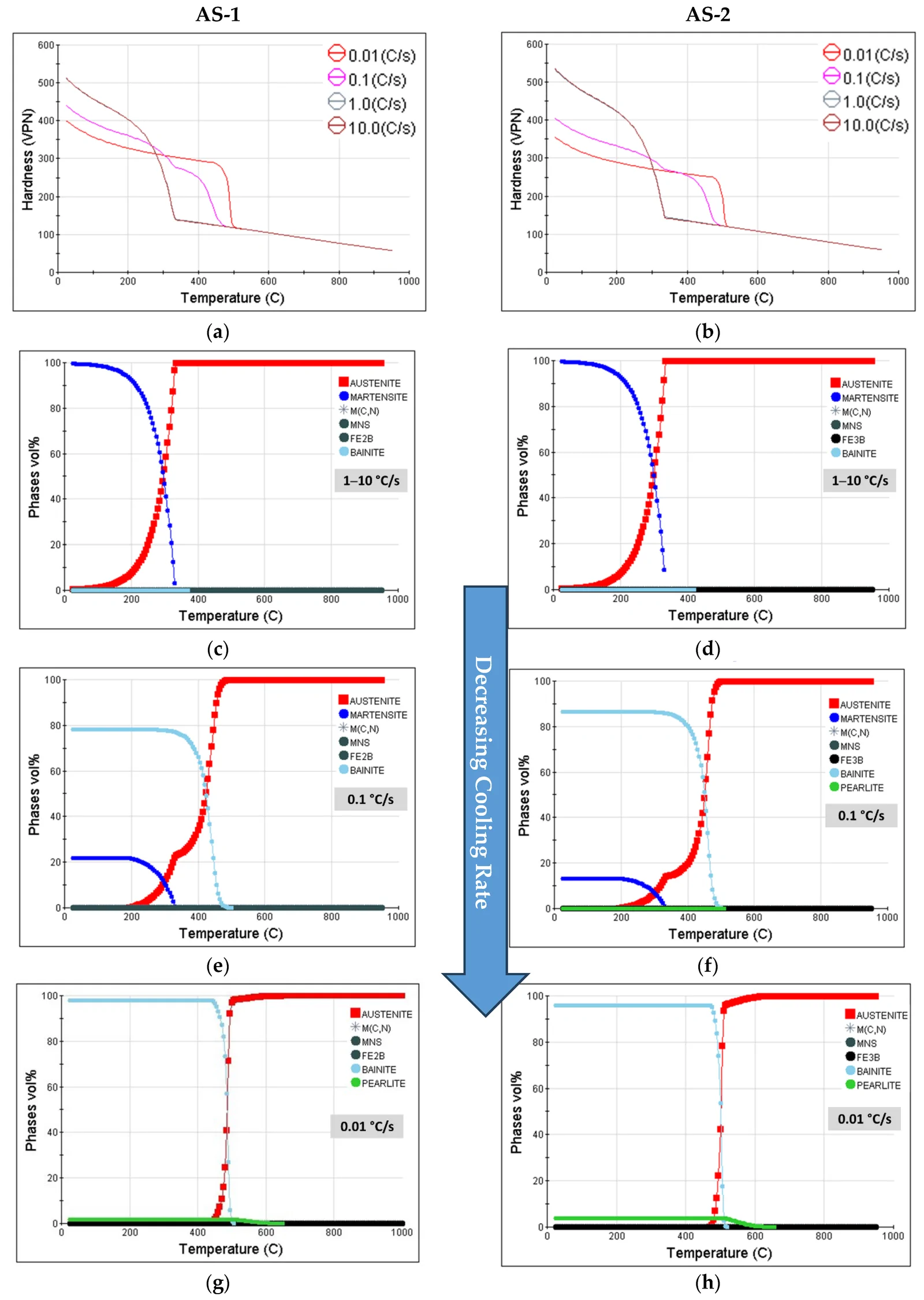
The JMATPro curves show the cooling rate effect on phases and hardness: (**a**) hardness of AS-1, (**b**) hardness of AS-2, (**c**) AS-1 at 1–10 °C/s, (**d**) AS-2 at 1–10 °C/s, (**e**) AS-1 at 0.1 °C/s, (**f**) AS-2 at 0.1 °C/s, (**g**) AS-1 at 0.01 °C/s, and (**h**) AS-2 at 0.01 °C/s. * M(C, N): where M could be Fe, Ni, Mo, Nb, Si, Cr, and Mn; MNS: manganese sulphide inclusions; FE2B and FE3B: iron boron precipitates.

From Figure 1, by employing JMATPro (grain size: 9.0 ASTM, austenitization: 950 °C), it was found that in AS-1, when the cooling rate increased, the hardness increased until it reached the highest value of 1 °C/s, due to the formation of full martensite (500 HV); higher cooling rates of more than 1 °C/s no longer affected the hardness. However, when the cooling rate was less than 1 °C/s, the hardness decreased because of the increment of bainite, with 450 HV (80% bainite) at 0.1 °C/s and 400 HV (98% bainite) at 0.01 °C/s. Pearlite was only formed at the lowest cooling rate of 0.01 °C/s. Furthermore, when increasing the Nb and B in AS-2, the hardness increased to 550 HV with high cooling rates of 1 to 10 °C/s, while the hardness decreased with low cooling rates, by 400 HV (87% bainite) at 0.1 °C/s and 350 HV (98% bainite) at 0.01 °C/s, because of the increment of bainite and pearlite. Therefore, it is essential to improve hardenability by suppressing the formation of high-temperature transformation products like ferrite, pearlite, and bainite and by promoting martensite formation.

### 3.2. Phase Transformations of Nb-alloyed Armor Steel

**Figure 2:**
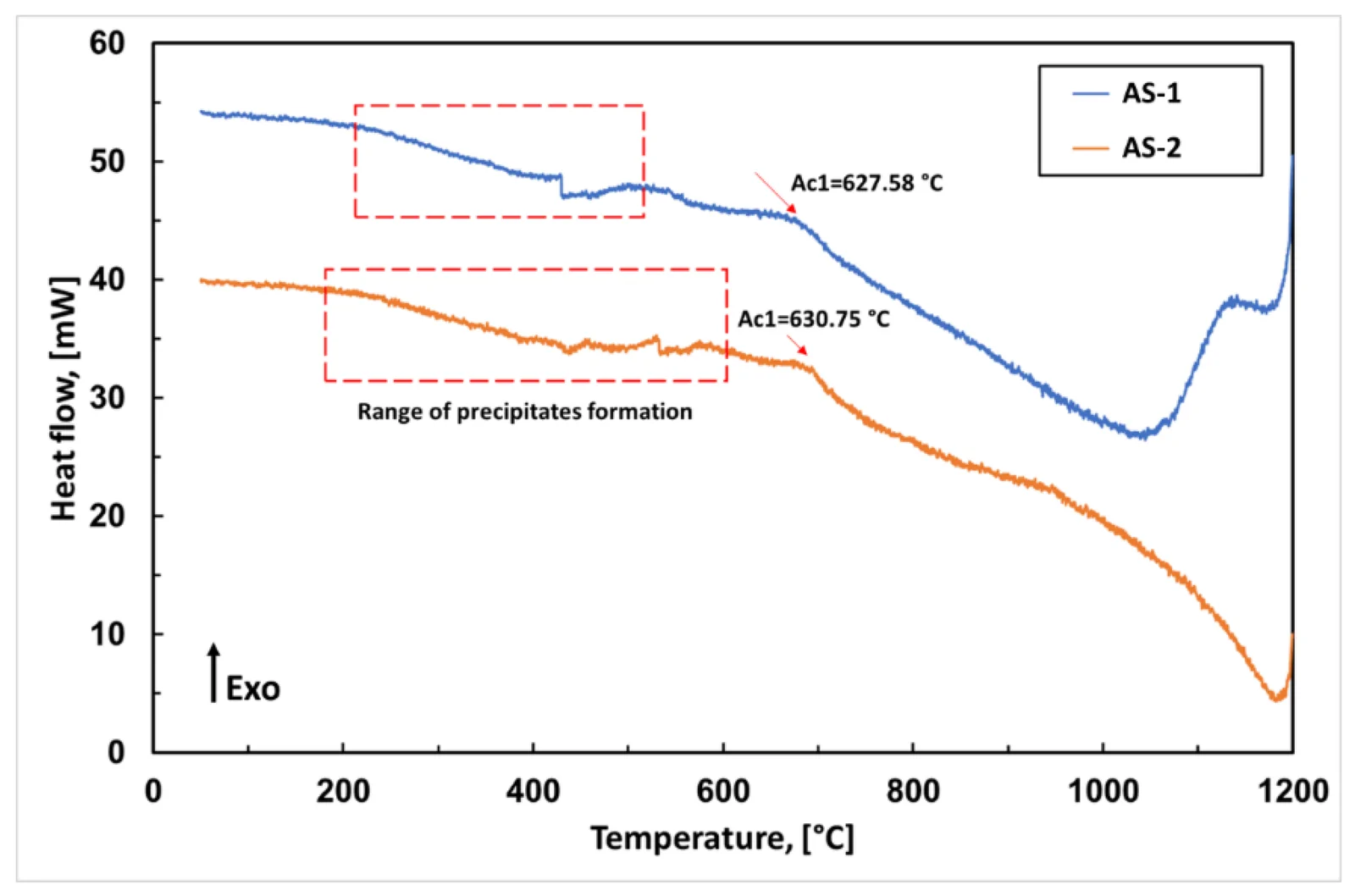
Offset DSC records during cooling from 1200 °C at a rate of 10 °C/min for both AS-1 and AS-2 alloys. The range of precipitates formation were marked in red box.

According to the DSC cooling curves shown in Figure 2, it was observed that there were several peaks in both AS-1 and AS-2. At low temperatures between 190 and 500 °C, a change in the DSC signal is evident, indicating the formation of carbides. However, in the AS-2 alloy, more peaks appeared during cooling than were present in AS-1. These transformation temperatures agree with the theoretical values obtained from the Thermo-Calc predictions published in our previous work [41]. Additionally, the Ac1 temperatures, measured at about 627 °C in AS-1 and 631 °C in AS-2, were found to be in good agreement with the ThermoCalc predictions and dilatation curves published in the same work [41].

### 3.3. Phases and Carbide Formation

**Figure 3:**
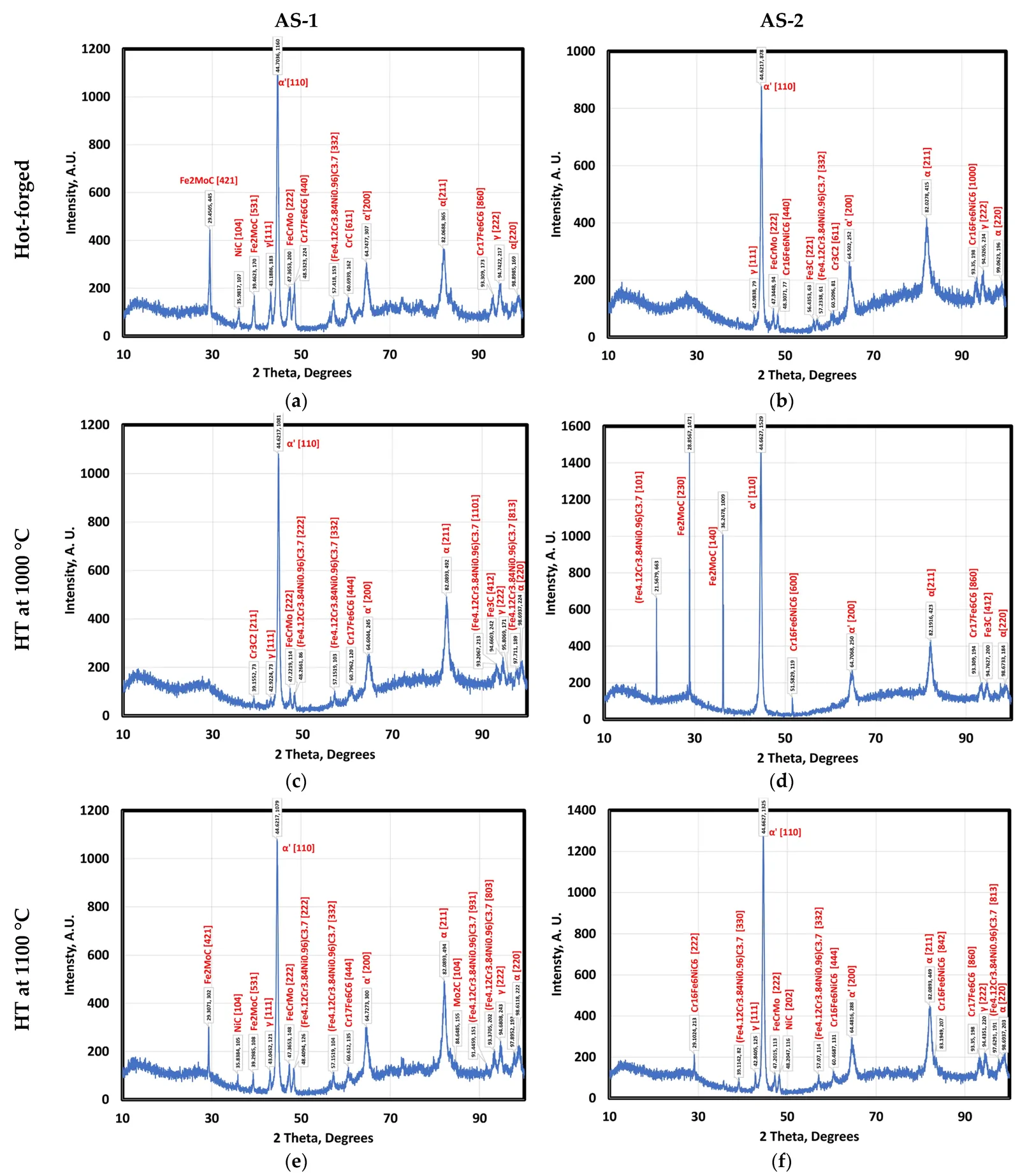
XRD curves of: (**a**) hot-forged AS-1, (**b**) hot-forged AS-2, (**c**) AS-1 HT at 1000 °C, (**d**) AS-2 HT at 1000 °C, (**e**) AS-1 HT at 1100 °C, and (**f**) AS-2 HT at 1100 °C.

The XRD analysis, shown in Figure 3, exhibits the distinct peaks of martensite, austenite, ferrite, and carbides in the hot-forged and heat-treated (HT) Nb microalloyed steels. Various carbides, such as Fe, Mo, Ni, and Cr carbides, were found in the developed alloys. These carbides were predicted in ThermoCalc as cementite and the KSI carbides (Fe, Cr, Mo)3(C)1, M3C2, MC, M7C3, M23C6, and M6C, where M could be Fe, Ni, Mo, Nb, Si, Cr, and Mn [41].

### 3.4. Microstructural Investigations

**Figure 4:**
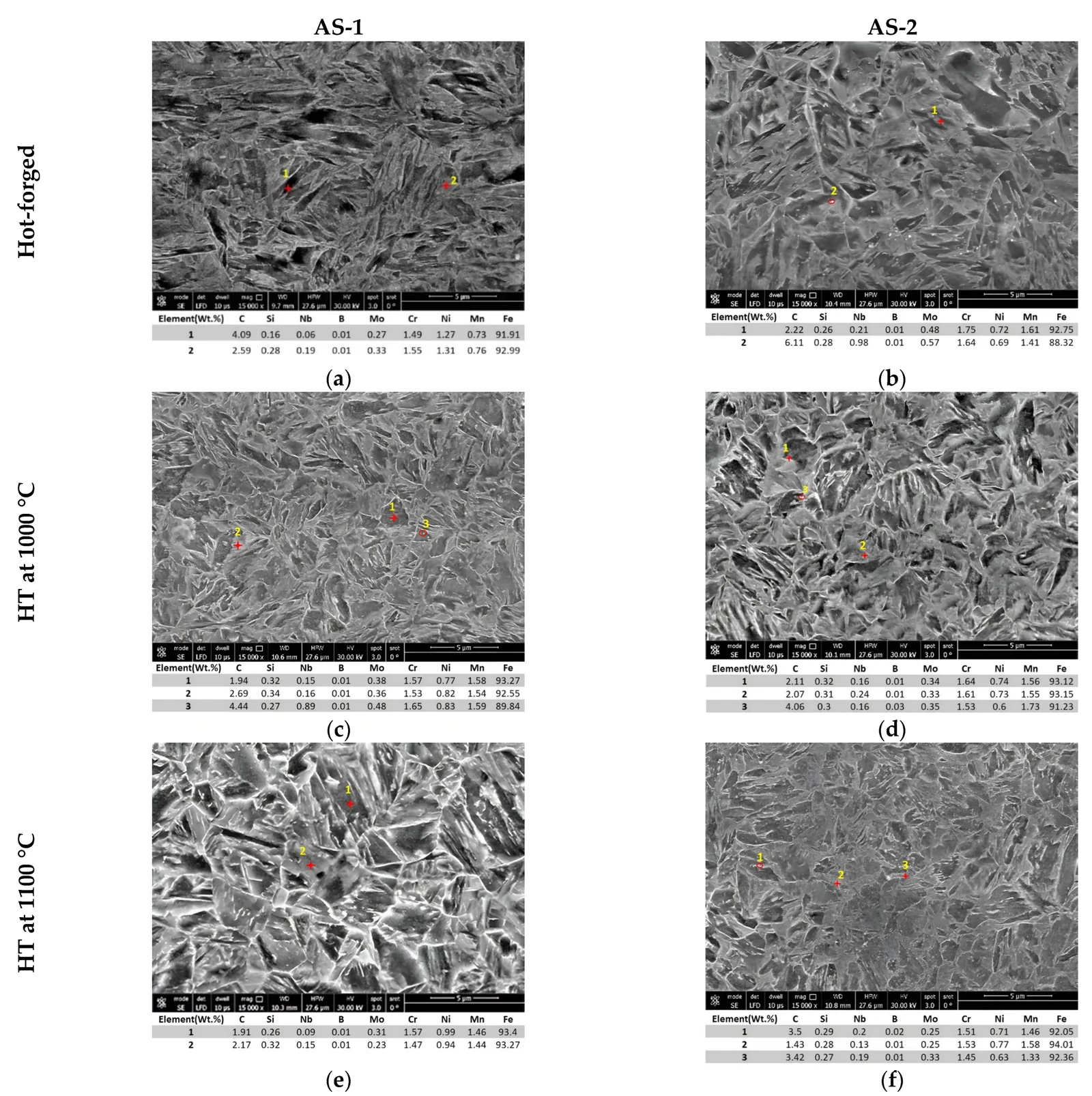
SEM with EDX analysis: (**a**) hot-forged AS-1, (**b**) hot-forged AS-2, (**c**) AS-1 HT at 1000 °C, (**d**) AS-2 HT at 1000 °C, (**e**) AS-1 HT at 1100 °C, and (**f**) AS-2 HT at 1100 °C.

Figure 4 shows the SEM microstructure of the produced steel via EDX analysis of the martensitic structure, demonstrating homogeneous distributed Nb carbides with roundish and capsular shapes precipitated at the grain boundaries. Martensitic laths were observed with different shapes: short, long, blocky, and equiaxed shapes.

### 3.5. Evaluation of Phases Ratios and Surface Texture

**Figure 5:**
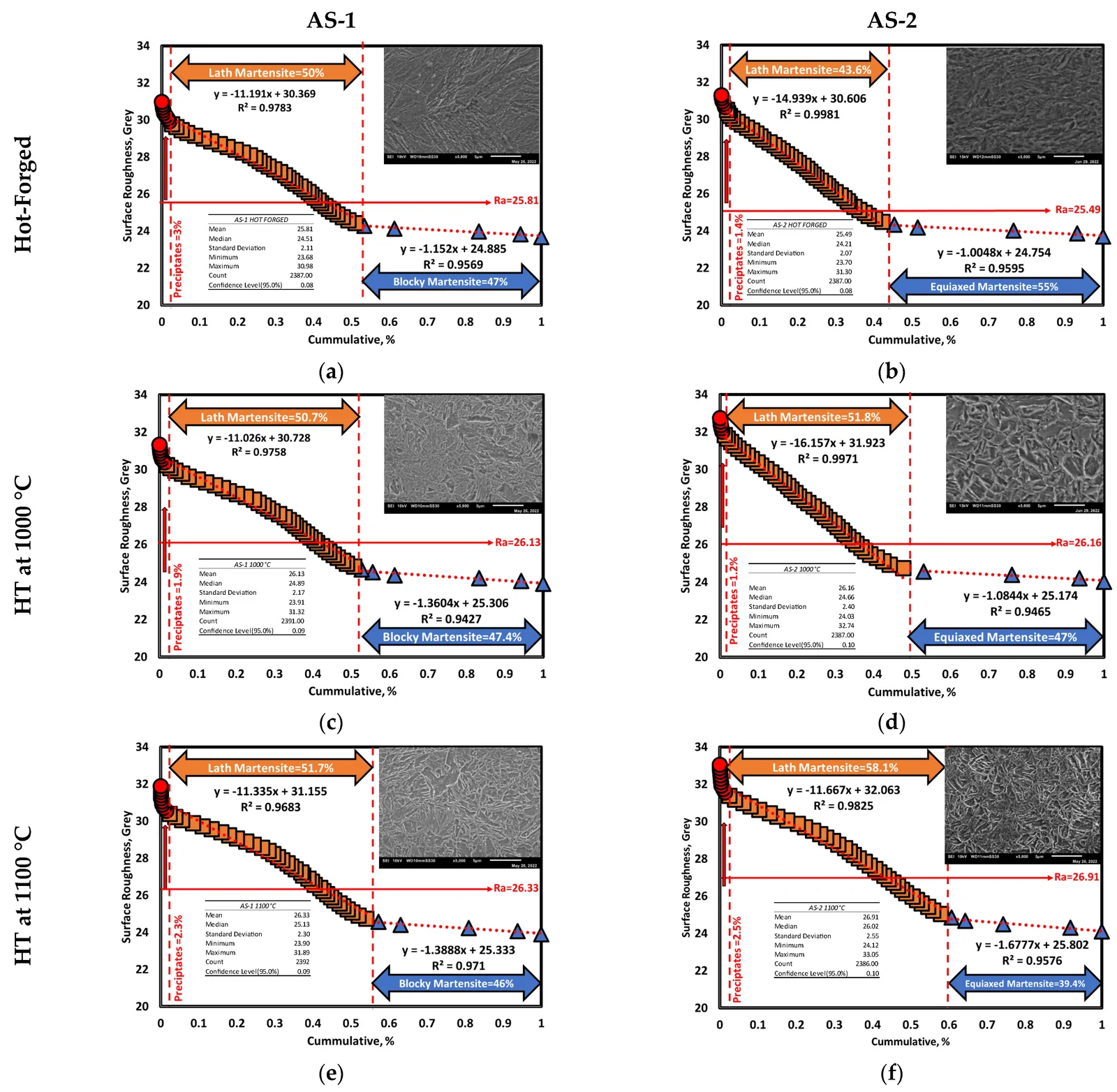
Abbott–Firestone curves: (**a**) hot-forged AS-1, (**b**) hot-forged AS-2, (**c**) AS-1 HT at 1000 °C, (**d**) AS-2 HT at 1000 °C, (**e**) AS-1 HT at 1100 °C, and (**f**) AS-2 HT at 1100 °C.

MATLAB R2023a software was utilized to generate the Abbott–Firestone curves based on the SEM surface texture images of Nb microalloyed Armor steel samples, as depicted in Figure 5a–f. The Abbott–Firestone curves are a valuable tool for assessing the number and ratio of phases from SEM surface texture images. These curves cluster the surface texture image into three groups of peaks: high peaks (representing precipitates), mean peaks (indicating lath martensite carrying the load), and low peaks (referring to equiaxed/blocky martensite), according to ISO 25178-2:2021 [45].

Figure 5c,e shows that after heat treatment of the AS-1 sample, the ratio of lath martensite slightly increased by 1.7%, while blocky martensite decreased by 1%. In the case of the hot-forged AS-2 samples, the long-lath martensite and blocky martensite transformed into short-lath and equiaxed martensite (which is relatively fine), as is evident in the SEM images in Figure 5b. Furthermore, after the heat treatment of AS-2, the ratio of short-lath martensite increased by 14.5%, while equiaxed martensite decreased by 15.6%, as shown in Figure 5d,f. Additionally, in hot-forged AS-2, the percentage of precipitates decreased by 1.6% compared to hot-forged AS-1, due to the grain refinement resulting from the increased Nb content, which suppresses the formation of NbC precipitates and reduces the precipitation ratio. However, the precipitate percentages are nearly equal in both AS-1 and AS-2 after each heat treatment at 1000 °C and 1100 °C. These results were in agreement with the grain size calculations published in our previous work [41].

**Figure 6:**
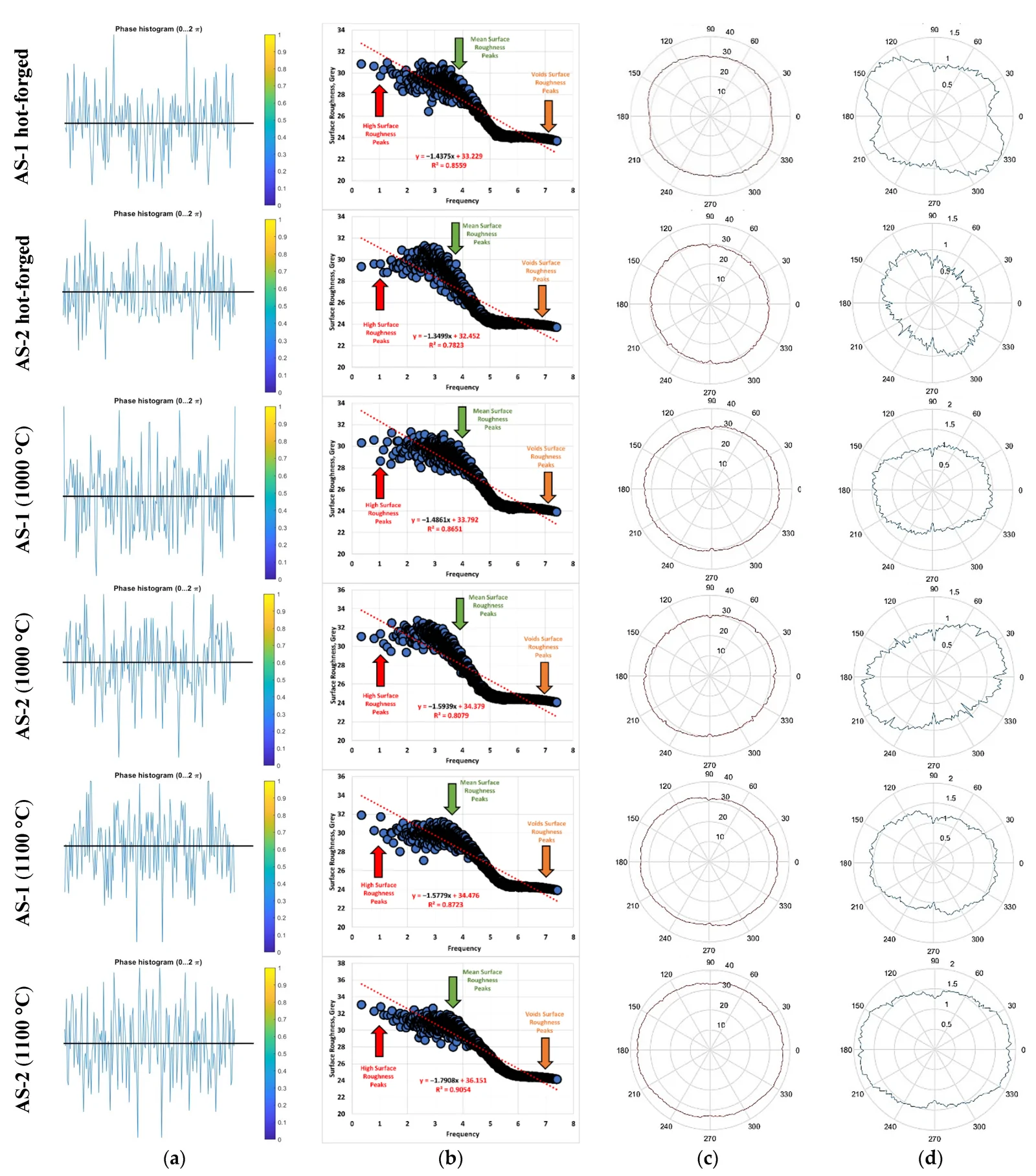
(**a**) Surface roughness histogram, (**b**) surface texture, (**c**) intercept rose plot, and (**d**) slope rose plot of the investigated Armor alloys.

Based on the Abbott–Firestone analysis, Figure 6a–d illustrates the corresponding surface roughness, surface texture, intercept rose, and slope rose of the SEM images of AS-1 and AS-2 alloys after hot forging and heat treatment.

Figure 6a,b presents the qualitative surface roughness and texture results. Low surface roughness values correspond to blocky/equiaxed martensite, moderate surface roughness values indicate long/short lath martensite, and high surface roughness values signify the precipitates.

Furthermore, Figure 6c,d presents the intercept rose and slope rose distribution, respectively, in a polar plot format, with negligible distortion in AS-2 and complete distortion in AS-1. However, all samples exhibited regular fluctuations in the surface texture, except for the hot-forged AS-1 sample.

To validate the Abbott–Firestone calculations regarding phase ratios and surface texture, tribological tests were conducted to evaluate the wear rate, friction coefficient, and surface roughness.

### 3.6. Tribological Investigations

**Figure 7:**
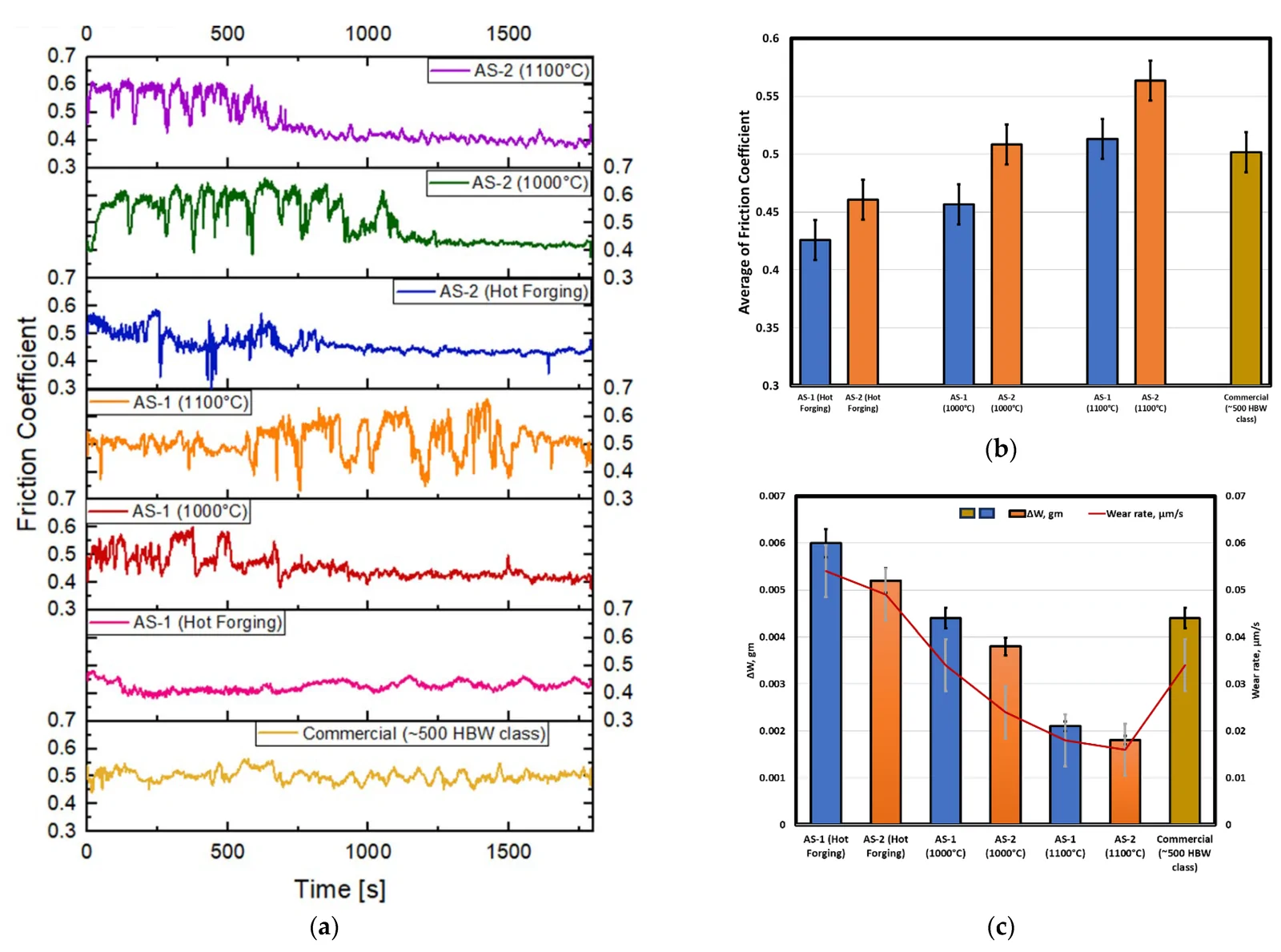
The effect of Nb microalloying and austenite grain refinement on the tribological characteristics of the investigated Armor alloys: (**a**) friction-versus-time plots, (**b**) the average values of the friction coefficient, (**c**) wear rate, and wear loss (∆W).

**Figure 8:**
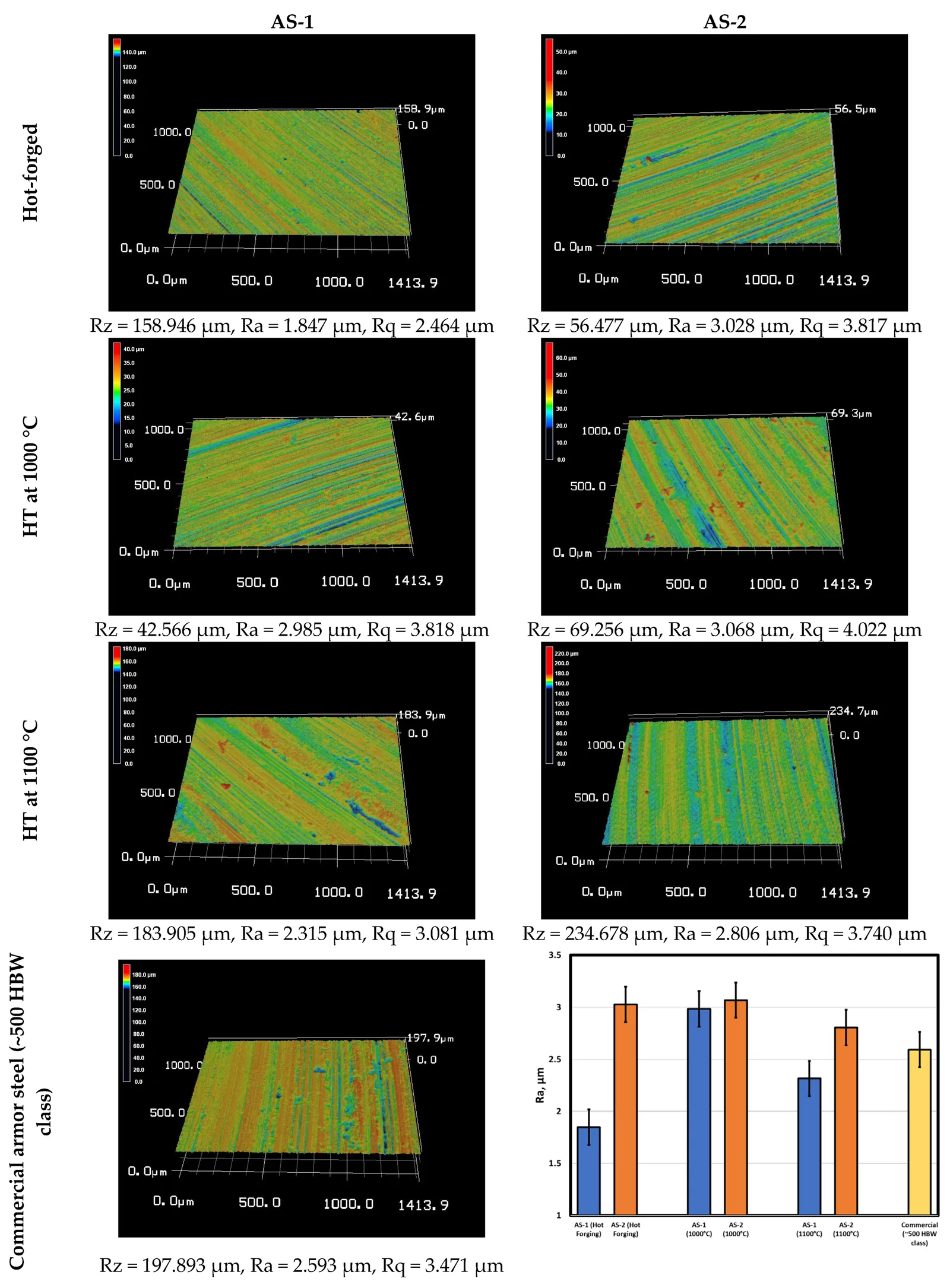
Worn surfaces after wear tests: 3D laser scanning images of the investigated Armor alloys, indicating the roughness parameters of mean roughness depth (Rz), root mean square roughness (Rq), and mean roughness (Ra) values.

From Figure 7, it can be seen that the friction coefficient increases with the microalloying of Nb content and austenite grain refinement. To better understand the wear mechanisms that were present in the investigated Armor alloys, an analysis of the worn surfaces was conducted using a 3D laser scanning microscope (Keyence VK-X100) (Keyence Corporation of America, Elmwood Park, NJ, USA), as seen in Figure 8. Similar surface wear characteristics were discovered in both alloys, which comprised smooth and thin grooves. AS-1 alloy exhibited evident surface wear that was characterized by uninterrupted scratches, suggesting significant plastic deformation being caused by the harder counter-face of the wear machine disc. The formation of shallower wear tracks indicates that the austenite refinement heat treatment and the increased Nb in the AS-2 alloy significantly impacted its wear characteristics. This observation indicates an enhancement of the hardness and strength, hence emphasizing the beneficial influence of Nb additions and austenite refinement cycles on the overall resistance to wear.

### 3.7. Microhardness

**Figure 9:**
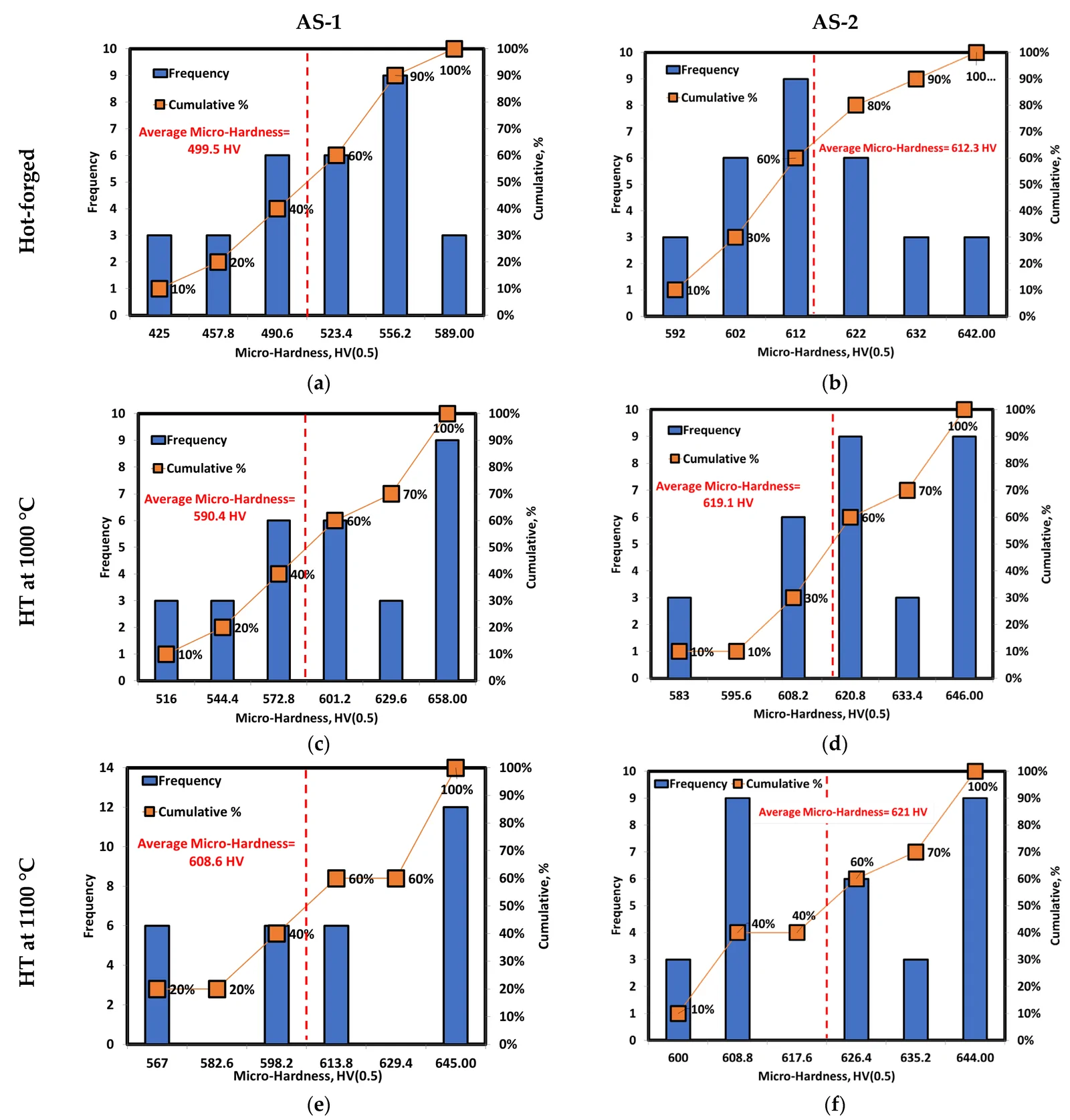
Average micro-hardness (HV 0.5) of the investigated Armor alloys: (**a**) hot-forged AS-1, (**b**) hot-forged AS-2, (**c**) AS-1 HT at 1000 °C, (**d**) AS-2 HT at 1000 °C, (**e**) AS-1 HT at 1100 °C, and (**f**) AS-2 HT at 1100 °C.

Figure 9 shows the measured microhardness values of the investigated Armor alloys. It was found that the average hardness in hot-forged AS-1 is 500 (HV 0.5); this value was increased to nearly 600 (HV 0.5), either by increasing the Nb content or the austenite refinement heat treatment cycle. In their previously published paper [41], the authors mentioned that the measured Brinell hardness values confirmed the positive impact of Nb and heat treatment on the refinement of the produced martensitic structure. The hardness of the AS-1 and AS-2 alloys has been increased by 14% compared to the conventional Armor Steel.

## 4. Conclusions

The effect of Nb microalloying and austenite grain refinement using a specific heat treatment on Armor steel was investigated. The following conclusions could be drawn:

- JMATPro software (version 13.2) analysis revealed that in order to enhance hardenability, it is crucial to control the cooling rate to suppress the formation of high-temperature transformation products such as ferrite, pearlite, and bainite while promoting the formation of martensite. The results showed that at high cooling rates above 1 °C/s, the microstructures of AS-1 and AS-2 were fully martensitic, resulting in hardness values of 500 HV and 550 HV, respectively.
- The predicted and measured phase transformations identified several transformation temperatures (peaks), including Ac1, and the precipitation of carbides such as M3C2, MC, M7C3, M23C6, M6C, cementite, and KSI carbides. These phases were indicated in the XRD curves and thSEM microstructure via EDX analysis.
- Surface texture characterization using the Abbott–Firestone curve provided insights into the phase distribution. Three groups of peaks were identified: precipitates, short/long lath martensite, and equiaxed/blocky martensite. It was observed that when the Nb content was increased in AS-2, the long-lath martensite and blocky martensite transformed into short-lath and equiaxed martensite with a finer structure. Moreover, the short/long lath martensite content slightly increased while equiaxed/blocky martensite content decreased after heat treatment. Moreover, the percentage of precipitates decreased in AS-2, due to the grain refinement mechanism, which reduced the precipitation.
- The wear characteristics of the investigated alloys were evaluated using a pin-on-disc tribometer. The results demonstrate that alloying with Nb, along with grain refinement using a thermal cycle, significantly reduce the wear rate.
- These findings were validated using microhardness measurements, which demonstrated that the average hardness of hot-forged AS-1 was 500 (HV 0.5). This value increased to approximately 600 (HV 0.5) with the introduction of higher Nb content or through austenite refinement via heat treatment cycles. However, in the case of AS-2, the hardness values remained nearly the same after grain refinement via heat treatment cycles.
- In conclusion, the incorporation of Nb microalloying and austenite grain-refining heat treatment techniques in Armor steel manufacture showed promising results in terms of achieving improved hardness, phase distribution, surface texture, and tribological properties. These findings contribute to the development of enhanced lightweight armor steel with superior protective properties.

## Figures and Tables

**Table 1:** Chemical composition in wt % of the produced alloys.

Alloy No.	C	Si	Mn	Cr	Mo	Ni	Nb	B	P	S	Fe
**AS-1**	0.25	0.22	0.87	1.22	0.7	1.69	0.07	0.0035	0.013	0.008	Bal.
**AS-2**	0.28	0.18	0.70	1.21	0.64	1.64	0.13	0.0046	0.013	0.008	Bal.

## Data Availability

The datasets used and/or analyzed during the current study are available from the corresponding author upon reasonable request.
