# Correction: Youssef et al. The Influences of Nb Microalloying and Grain Refinement Thermal Cycling on Microstructure and Tribological Properties of Armor Steel. *Materials* 2023, *16*, 7485

**DOI:** 10.3390/ma19183834

**Published:** 2026-09-09

**Authors:** Mervat Youssef, Eman H. El-Shenawy, Wael Khair-Eldeen, Tadaharu Adachi, Adel Nofal, Mohsen A. Hassan

**Affiliations:** 1 School of Innovative Design Engineering (IDE), Egypt-Japan University of Science and Technology (E-JUST), Alexandria 21934, Egypt; wael.khaireldin@ejust.edu.eg; 2 Metal Casting Department, Manufacturing Technology Institute, Central Metallurgical Research and Development Institute (CMRDI), Cairo 11912, Egypt; adelnofal@hotmail.com; 3 Plastic Deformation Department, Metal Technology Institute, Central Metallurgical Research and Development Institute (CMRDI), Cairo 11912, Egypt; dremanelshenawy@cmrdi.sci.eg; 4 Department of Mechanical Engineering, Toyohashi University of Technology (TUT), Aichi 441-8122, Japan; adachi.tadaharu.or@tut.jp

In the original publication [1], there was an error in which the trademarked product name “Armox 500T/Armox” was used to describe the studied material. Corrections have been made throughout the paper.

The words “Armox 500T” are updated to “Armor Steel” in the Title, Keywords, Section 1 paragraph 9, and Section 3.7 paragraph 1.

The words “Armox 500T” are updated to “Armor” in Section 2.8 paragraph 1, the title of Section 3.2, Section 3.5 paragraph 1, and Section 4 paragraphs 1 and 7.

The word “Armox” is updated to “Armor” in Abstract; Section 1 paragraph 3, paragraph 8, and paragraph 9; Section 2.1 paragraph 1; Section 2.3; the captions of Figures 6–9; Section 3.6 paragraph 1; and Section 3.7 paragraph 1.

The words “Armox 1” and “Armox 2” are updated to “AS-1” and “AS-2” in Section 3.1 paragraph 1; Section 3.2 paragraph 1; Section 3.5 paragraphs 2, 3, and 5; Section 3.6 paragraph 1; Section 3.7 paragraph 1; Section 4 paragraphs 2, 4 and 6; Figures 1–9; the captions of Figures 1–5 and 9; and Table 1.

In the Abstract, the words “Armox 500T steel” are updated to “two newly developed armor steels prepared on a laboratory scale”; the words “Armox 500T alloy” are updated to “investigated alloys”.

In Section 1 paragraph 2, the words “Armox 500T steel” are updated to “Commercial quenched-and-tempered armor steel (≈500 HBW class)”; the words “Armox 500T plates” are updated to “The final plates”; in paragraph 8, the words “Armox 500T” are updated to “commercial quenched-and-tempered armor steel (~500 HBW class)”; in paragraph 9, the words “Armox 500T steel” are updated to “two newly developed armor steels prepared on a laboratory scale”.

The authors state that the scientific conclusions are unaffected. This correction was approved by the Academic Editor. The original publication has also been updated.
